# Editorial: Resolution of inflammation: Mechanisms, mediators and biomarkers: Volume II

**DOI:** 10.3389/fphar.2022.1100420

**Published:** 2022-11-24

**Authors:** Paola Patrignani, Bernhard Brüne, Dieter Steinhilber

**Affiliations:** ^1^ Department of Neuroscience, Imaging and Clinical Science, School of Medicine and CAST, G d’Annunzio University, Chieti, Italy; ^2^ Institute of Biochemistry I, Faculty of Medicine, Goethe University, Frankfurt, Germany; ^3^ Fraunhofer Institute for Translational Medicine and Pharmacology, ITMP and Fraunhofer Cluster of Excellence for Immune Mediated diseases, CIMD, Frankfurt, Germany; ^4^ Institute of Pharmaceutical Chemistry, Goethe University, Frankfurt, Germany

**Keywords:** lipoxygenase, SPM, resolution of inflammation, CD40, resveratrol, sphingolipids, NASH

Resolution of inflammation is now considered an active process, which is accompanied by a switch in the profile of cytokines, lipid mediators, and other signaling molecules. In addition to the alteration in the profile of the involved mediators, resolution of inflammation is also linked to cellular plasticity, which allows the polarization of immune cells from a pro-inflammatory to an anti-inflammatory cell type. A well-established example is the polarization of monocytes to pro-inflammatory M1-like macrophages and anti-inflammatory M2-like cells. These changes in macrophage polarization are accompanied by a switch in the formed lipid mediators, partly due to the slight downregulation of 5-lipoxygenase (5-LO) and 5-LO activating protein (FLAP) and the prominent upregulation of 12/15-LO. A current paradigm is that changes in macrophage polarization is associated with a change in the released lipid mediator profile from pro-inflammatory prostaglandins and leukotrienes to so-called specialized pro-resolving lipid mediators (SPMs) such as lipoxins and resolvins. A hallmark of lipoxins and resolvins is that their biosynthesis from arachidonic acid (AA), eicosapentaenoic acid (EPA), and docosahexaenoic acid (DHA) requires the consecutive action of 5-LO and 12/15-LO.

Schebb et al. critically review the formation, signaling, and analytics of SPMs. Since 5-LO is mainly expressed in leukocytes, these cells are considered the main source of SPMs. The review article summarizes the biosynthetic pathways of SPMs in polymorphonuclear leukocytes (PMNL) as well as in M1 and M2 macrophages. It was concluded that human leukocytes only have a very limited biosynthetic capacity for trihydroxylated SPMs (lipoxins, RvE1, RvD1, RvD2, RvD3, RvD4). Therefore, the formation of most of these SPMs is too low to be detected by liquid chromatography-mass spectrometry (LC-MS) in biological samples using the generally accepted peak definition based on the signal-to-noise ratio. Instead, a nonstandard method for peak assignment was introduced, allowing the inclusion of signal noise into the peak assignment. Furthermore, peak illustrations are shown in numerous SPM publications (commented in PubPeer) instead of the original chromatograms, so that the validity of these data is unclear at the moment. The review also summarizes the current knowledge on SPM receptors, and it was concluded that the assignment of the SPM ligand-receptor pairs is not validated at the moment and that the signal transduction elicited by many SPMs is still unclear. Besides these shortcomings, it is generally accepted that macrophage polarization to M2 leads to a lipid mediator switch from the 5-LO to the 15-LO pathway. However, the identity of the key lipid mediators that drive the resolution of inflammation is still a matter of debate.

Benatzy et al. review the current information on the second of the two 15-LOs (ALOX15B), highlighting the enzymatic function in catalyzing the synthesis of anti-inflammatory and pro-resolving lipid mediators. Unlike ALOX15, which produces both 12(S)- and 15(S)-HETE from AA, ALOX15B exclusively catalyzes the peroxidation at the C15 of AA. In addition to the formation of lipoxin precursor 15(S)-HETE, ALOX15B was also associated with the formation of precursors for resolvin D5 (RvD5) synthesis from DHA. Besides reviewing the enzymatic function, the authors emphasize the role of ALOX15B in cancer, where a tumor suppressor role was apparent for both prostate and breast, whilst expression and function in other carcinomas remains unclear. The article highlights the implication of ALOX15B and its products in altering cellular signaling pathways, including PPAR, MAPK, and PI3K/AKT in cancer and inflammatory diseases. The role of ALOX15B in macrophage chemokine and cytokine production was discussed from underlining experiments utilizing inhibitors for 15-lipoxygenating enzymes suggesting a role in the resolution of inflammation.

The role of the CD40L-CD40-TRAF signaling cascade in the regulation of macrophage polarization as well as in atherosclerosis and its potential link to resolution is reviewed by Strohm et al. As CD40L can activate different cellular receptors, it can induce a broad range of inflammatory processes that may be detrimental or beneficial. The review concluded that CD40(L)-based therapies may be Janus-faced and require sophisticated fine-tuning to promote cardioprotection. Therefore, targeting CD40 signaling on specific cells or at specific levels of the involved signaling cascades could be a promising strategy to reestablish tissue homeostasis after myocardial infarction.

The role of T cells in the resolution of inflammation is reviewed by Hartel et al. with a special focus on sphingolipids. The authors summarize how sphingolipids can interfere with the differentiation and activation process of T cells. The authors highlight that a particular sphingolipid membrane composition seems to be required in the different subsets of T cells for individual signaling. The authors conclude that sphingolipids can modulate the T cell activities in diverse chronic inflammatory diseases that are driven by Th1 or Th2 cell responses or by an imbalance between Th17 and Tregs and that they are important for the resolution of inflammation.

Dalbeni et al. review the role of platelets in non-alcoholic steatohepatitis (NASH). The authors emphasize that the accumulation of platelets in the liver, platelet adhesion, and activation can prime immunoinflammatory reactions in the liver and the activation of stellate cells. Recent data suggest that antiplatelet drugs may interrupt this cascade, prevent/improve NASH, and improve some metabolic alterations. The pathophysiology of inflammatory liver disease and the role of platelets are discussed in this review.

Tang et al. summarize the therapeutic efficacy of resveratrol for acute lung injury. The authors conducted a meta-analysis based on 17 studies published from 2005 to 2017. It was concluded that resveratrol treatment seems to be effective in reducing the severity of acute lung injury but that more animal studies and clinical trials are required to elucidate the therapeutic benefit fully.

